# Epigenetic stratification of head and neck cancer survivors reveals differences in lycopene levels, alcohol consumption, and methylation of immune regulatory genes

**DOI:** 10.1186/s13148-020-00930-5

**Published:** 2020-09-11

**Authors:** Laura Moody, Sylvia L. Crowder, Andrew D. Fruge, Julie L. Locher, Wendy Demark-Wahnefried, Laura Q. Rogers, Ashley Delk-Licata, William R. Carroll, Sharon A. Spencer, Molly Black, John W. Erdman, Hong Chen, Yuan-Xiang Pan, Anna E. Arthur

**Affiliations:** 1 Division of Nutritional Sciences, University of Illinois at Urbana–Champaign, Urbana, IL 61801 USA; 2 Department of Food Science and Human Nutrition, University of Illinois at Urbana–Champaign, 386A Bevier Hall, MC-182, 905 South Goodwin Avenue, Urbana, IL 61801 USA; 3 Department of Nutrition, Dietetics, and Hospitality Management, Auburn University, Auburn, AL 36849 USA; 4 Department of Medicine, University of Alabama at Birmingham, Birmingham, AL 35294 USA; 5 Department of Nutrition Science, University of Alabama at Birmingham, Birmingham, AL 35294 USA; 6 Department of Otolaryngology, University of Alabama at Birmingham, Birmingham, AL 35294 USA; 7 Department of Radiation Oncology, University of Alabama at Birmingham, Birmingham, AL 35294 USA; 8 Illinois Informatics Institute, University of Illinois at Urbana–Champaign, Urbana, IL 61801 USA; 9 Carle Cancer Center, Carle Foundation Hospital, Urbana, IL 61801 USA

**Keywords:** Head and neck cancer, Survivors, DNA methylation, Inflammation, Lycopene, Alcohol

## Abstract

**Background:**

Inflammation has been associated with higher rates of recurrence and mortality in head and neck cancer (HNC). While the biological mechanisms predisposing patients to heightened inflammatory states remain largely unknown, DNA methylation has been proposed to reflect systemic inflammation. In this analysis, we attempt to identify meaningful epigenetic patterns in HNC survivors by stratifying individuals based on DNA methylation profiles in leukocytes.

**Results:**

We used hierarchical clustering to uncover three distinct methylation patterns among HNC survivors. Each group displayed a unique methylation signature in inflammatory pathways including cytokine and B-cell receptor signaling. Additionally, we examined physiological, clinical, and lifestyle parameters related to inflammation, such as circulating carotenoid and cytokine levels, cancer treatment type, and alcohol consumption. Specifically, we identified one group of survivors who had significant differential methylation of transcriptional and translational regulators as well as genes in the T-cell receptor signaling pathway, including hypermethylation of CD40 ligand (*CD40LG*) and Tec protein tyrosine kinase (*TEC*) and hypomethylation of *CD8A*. This group also displayed high circulating lycopene levels. We identified another group that had distinctive methylation in the toll-like receptor (TLR) signaling pathway, including hypomethylation of *TLR5*, a component of the inhibitor of nuclear factor-kappa B kinase complex (*CHUK*), and two mitogen-activated protein kinases (*MAP3K8* and *MAP2K3*). This group also had hypermethylation of mitochondrial ribosomal genes along with higher rates of alcohol consumption.

**Conclusion:**

The correlation between lycopene, alcohol consumption, DNA methylation, and inflammation warrants further investigation and may have implications in future recommendations and interventions to impact health outcomes in HNC survivors.

## Introduction

Cancer is driven by genomic instability that impacts cell growth, metabolism, and inflammation. As the immune system attempts to suppress tumor proliferation, tumor cells adapt to evade immune recognition and inhibit the immune response. Furthermore, inflammatory processes play a role in the creation of the tumor microenvironment as well as impact long-term patient outcomes [1, 2]. Inflammatory and bioactive compounds also interfere with epigenetic processes [3, 4]. Previous studies have found that carotenoid intake and plasma concentration are associated with DNA methylation in blood leukocytes, especially surrounding inflammatory genes [5, 6]. This is of particular interest, considering that uncontrolled chronic inflammation can induce tumorigenesis via the growth factor activity of cytokines as well as persistent production of reactive oxygen species [7, 8]. It is currently unknown whether circulating levels of cytokines and carotenoids induce beneficial epigenetic modifications, but evidence suggests that modulation of such compounds may be used in cancer prevention and therapeutics [9]. For instance, lycopene has been shown to reduce oxidative stress [10] and demethylate tumor suppressor genes in vitro [4]. In a large prospective cohort study, lycopene supplementation was also associated with a lower risk of cancer mortality [11]. Thus, understanding the relationship between inflammation, dietary factors, the epigenome, and cancer progression can help to prevent recurrence and improve survival and quality of life in cancer survivors.

Head and neck cancer (HNC) survivors are a particularly vulnerable population that may benefit greatly from advances in immunology and epigenetics research. HNC survivors are often faced with persistent symptoms, such as difficulty swallowing (dysphagia), dry mouth (xerostomia), and taste alterations [12–15]. Survivors may also experience inflammatory issues, including lymphedema, ulceration of the mucosal membranes, and increased susceptibility to infection. Not only do HNC patients have higher cytokine levels than controls, but cytokine levels continue to remain high even after radiation and chemoradiation therapy [16–18]. Higher levels of interleukin 6 (IL-6) and C-reactive protein (CRP) have been associated with higher levels of fatigue after radiation [19]. Interestingly, inflammatory biomarkers can also predict patient survival and recurrence. Elevated levels of IL-6 and high neutrophil-to-lymphocyte ratio have been linked to greater recurrence and shorter overall survival [20, 21]. Human papillomavirus (HPV) status of tumors also appears to create a distinct inflammatory landscape which dictates epigenetic markers and favorable prognosis [22–24]. Another study used DNA methylation as a surrogate measure for systemic inflammation which could also be used to predict overall survival [25]. More evidence is needed to validate the inflammatory processes that are correlated with epigenetic remodeling. This may enable healthcare providers to monitor recurrence and general health, as well as offer optimal counseling to HNC survivors.

Herein, we report an analysis of data collected as part of a pilot clinical trial in HNC survivors [26]. We investigate blood samples with the objective of characterizing survivors according to DNA methylation signatures in leukocytes. We also attempt to uncover the relationship between DNA methylation and inflammation by measuring circulating carotenoid and cytokine levels and quantifying lifestyle parameters such as alcohol consumption and smoking status. By elucidating the interplay between epigenetics and inflammation in HNC survivors, we hope to uncover specific epigenetic biomarkers of survivor health.

## Methods

### Study population and procedures

Participants were HNC survivors who had been treated previously at the University of Alabama-Birmingham (UAB) NCI-designated Comprehensive Cancer Center. Eligible participants were identified via the UAB Comprehensive Cancer Center Cancer Registry and recruited to participate in a pilot randomized clinical trial testing the feasibility of a 12-week dietary intervention focused on increasing cruciferous and green leafy vegetables. HNC survivors were eligible for participation if they were previously diagnosed with stage I–IV oral, pharyngeal, or laryngeal cancer, ≥6 months post-treatment and able to consume at least soft foods orally. Originally, 24 participants were recruited, but only 23 are included in this analysis due to missing serum cytokine and carotenoid data for one participant. A detailed description of the pilot study methods and population have been detailed elsewhere [26]. The study was approved by the Institutional Review Board at UAB and University of Illinois Urbana-Champaign (UIUC) and all participants provided written informed consent before initiating study activities.

### Blood collection

Whole blood (8.5 mL serum separator tube and 4 mL EDTA-treated tube) was drawn via venipuncture by a trained phlebotomist at the UAB Clinical Research Unit. Participants were asked to avoid smoking, alcohol, and exercise for 24 h prior to collection primarily because of associated acute effects on serum cytokines [27–29]. Blood was centrifuged between 1000 and 2000 *g*. Four 0.5 mL serum aliquots were prepared and stored at −80 °C until batch-analysis for cytokines and carotenoids. The buffy coat was extracted from the EDTA-treated tube for DNA methylation analysis.

### Serum proinflammatory cytokines

Serum cytokines (interferon-gamma (IFN- γ), interleukin-1 beta (IL-1β), IL-6, and tumor necrosis factor-alpha (TNF-α)) and CRP were assayed in the UAB Metabolism Core using MSD imager (MesoScale Discovery, Gaithersburg, MD; chemiluminescence technology; multiplex platform).

### Serum carotenoids

The analysis was performed under yellow lights to minimize light damage of carotenoids. Approximately 250 μL serum samples were mixed with an equal volume of ethanol containing 0.1% BHT and were vortexed for 30 s. One mL of hexane was added, vortexed, and centrifuged at 2400 rpm at 4 °C for 3 min. The hexane extraction step was repeated 2 times, and extracts were combined and dried under argon before being reconstituted in mobile phase for HPLC analysis. The extracts were separated on a reverse-phase C 30 column (4.6 × 150 mm, 3 μm; YMC, Wilmington, NC, USA) maintained at 18 °C. The gradient method used for carotenoid separation was based on the method of Yeum et al. [30]. All analyses were performed on an Alliance HPLC system (e2695 Separation Module) equipped with 2998 photodiode array detector (Waters, Milford, MA, USA).

### DNA methylation

Genomic DNA was extracted from whole blood using the DNA Purification from Buffy Coat protocol from the Gentra Puregene Blood Kit (Qiagen, Hilden, Germany). DNA methylation was measured via the HumanMethylation 450 BeadChip Array (Illumina, San Diego, CA, USA). Microarray data analysis was performed in R using the minifi package. To ensure acceptable sample quality, a detection *p* value was calculated for each sample based on the number of failed probes, and only samples with significant *p* values were included in the analysis. Next, stratified quantile normalization was performed. A probe was removed if it had a SNP at a CpG site or failed in at least one sample. The probes were annotated according to the hg19 genome.

### Statistical analysis

DNA methylation patterns were analyzed using a heuristic approach. To determine patterns in DNA methylation, hierarchical clustering with complete linkage was performed to stratify participants by methylation profile. Hierarchical clustering revealed three distinct methylation groups; they are designated with color yellow, blue, and pink, in subsequent results and figures. Further analysis of these groups was performed using the limma package to make pairwise comparisons. M-values were used to conduct analysis and report methylation values for differentially methylated probes (DMPs) and differentially methylated regions (DMRs) [31]. DMPs represent differential methylation at single CpGs, while DMRs represent clusters of consecutive differentially methylated CpGs [32]. A false discovery rate (FDR) adjusted *p* value <0.05 was considered significant.

Gene ontology enrichment analysis for molecular function and biological processes was performed using DAVID version 6.8 [33, 34]. Enrichment scores with FDR adjusted *p* values <0.05 were considered statistically significant. The top pathways were then ranked based on fold enrichment.

For analysis of circulating carotenoid and cytokine levels, Student’s *t* tests were used for pairwise comparisons. To test for differences between 3 groups, ANOVA was used to test for a group effect, and a post hoc Tukey test was performed to make pairwise comparisons. To test for group differences in the distribution of participants according to clinical and lifestyle characteristics, chi-square tests were performed. Alcohol and smoking were defined as self-report of consumption or use in the past 12 months for current and > 12 months for former. All statistical analyses were performed in R (version 3.4; R Foundation for Statistical Computing, Vienna, Austria).

## Results

### HNC survivors can be stratified according to three distinct methylation patterns

**Fig. 1:**
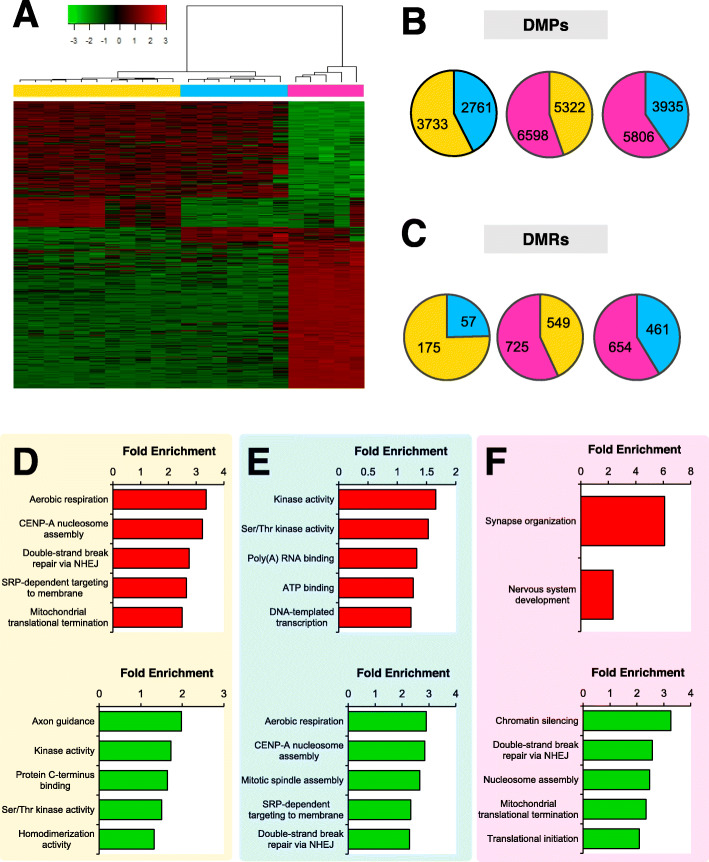
Three distinct methylation patterns were detected in blood samples of HNC survivors. **a** Representative heatmap shows the top DMPs with pairwise FDR *p* values <0.01 between the three clusters. **b** The pie charts show the number of differentially methylated probes and (**c**) differentially methylated regions within each of the comparisons (FDR *p* value <0.05). The numbers within the pie charts denote hypermethylated CpGs in the corresponding colored group. Gene ontology enrichment analysis was performed on the gene-associated DMPs in D) the yellow, E) blue, and F) pink methylation groups. Red graphs depict the analysis of hypermethylated DMPs and green graphs show enrichment in hypomethylated CpGs

DNA methylation was measured in blood samples collected from HNC survivors. Hierarchical clustering based on DNA methylation revealed three distinct groups (Fig. 1a). The groups are designated with colors yellow, blue, or pink. Pairwise comparisons between the heuristic groups revealed differentially methylated probes (DMPs) and differentially methylated regions (DMRs) (FDR adjusted *p* value <0.05; Fig. 1b, c). All DMPs and DMRs between each of the three pairwise group comparisons (blue vs pink, blue vs yellow, and pink vs yellow) are listed in Supplementary files 1, 2, 3, 4, 5 and 6.

Gene ontology enrichment analysis was performed to identify enriched biological processes and molecular functions. For each methylation group, gene-associated DMPs were divided by methylation status. In the yellow group, we first considered hypermethylated DMPs, which consisted of the 3733 DMPs that were hypermethylated compared withthe blue group and the 5322 DMPs that were hypermethylated compared with the pink group. Aerobic respiration, centromere protein A (CENP-A) nucleosome assembly, double-strand break repair via non-homologous end-joining (NHEJ), signal recognition particle (SRP)-dependent targeting to membrane, and mitochondrial translational termination were significantly enriched for hypermethylated DMPs (Fig. 1d). We then considered DMPs that were hypomethylated in the yellow group, which included the 2761 DMPs that were hypomethylated compared with the blue group and the 6598 that were hypomethylated compared with the pink group. Axon guidance, kinase activity, protein C-terminus binding, serine/threonine kinase activity, and homodimerization activity were enriched for hypomethylated DMPs (Fig. 1d). Similar analysis of the blue group revealed hypermethylation of kinase activity, serine/threonine kinase activity, poly(A) RNA binding, ATP binding, and DNA-templated transcription; and hypomethylation of aerobic respiration, CENP-A nucleosome assembly, mitotic spindle assembly, SRP-dependent targeting to membrane, and double-strand break repair via NHEJ (Fig. 1e). In the pink group, synapse organization and nervous system development were significantly enriched for hypermethylated DMPs, while chromatin silencing, double-strand break repair via NHEJ, nucleosome assembly, mitochondrial translation termination, and translation initiation were enriched for hypomethylated DMPs (Fig. 1f). For each analysis, the top five gene ontology categories are reported. All enrichment categories are specified in the Supplementary files 7, 8 and 9.

### Methylation patterns correspond with lycopene levels, alcohol intake, and epigenetic changes in immune pathways

**Fig. 2:**
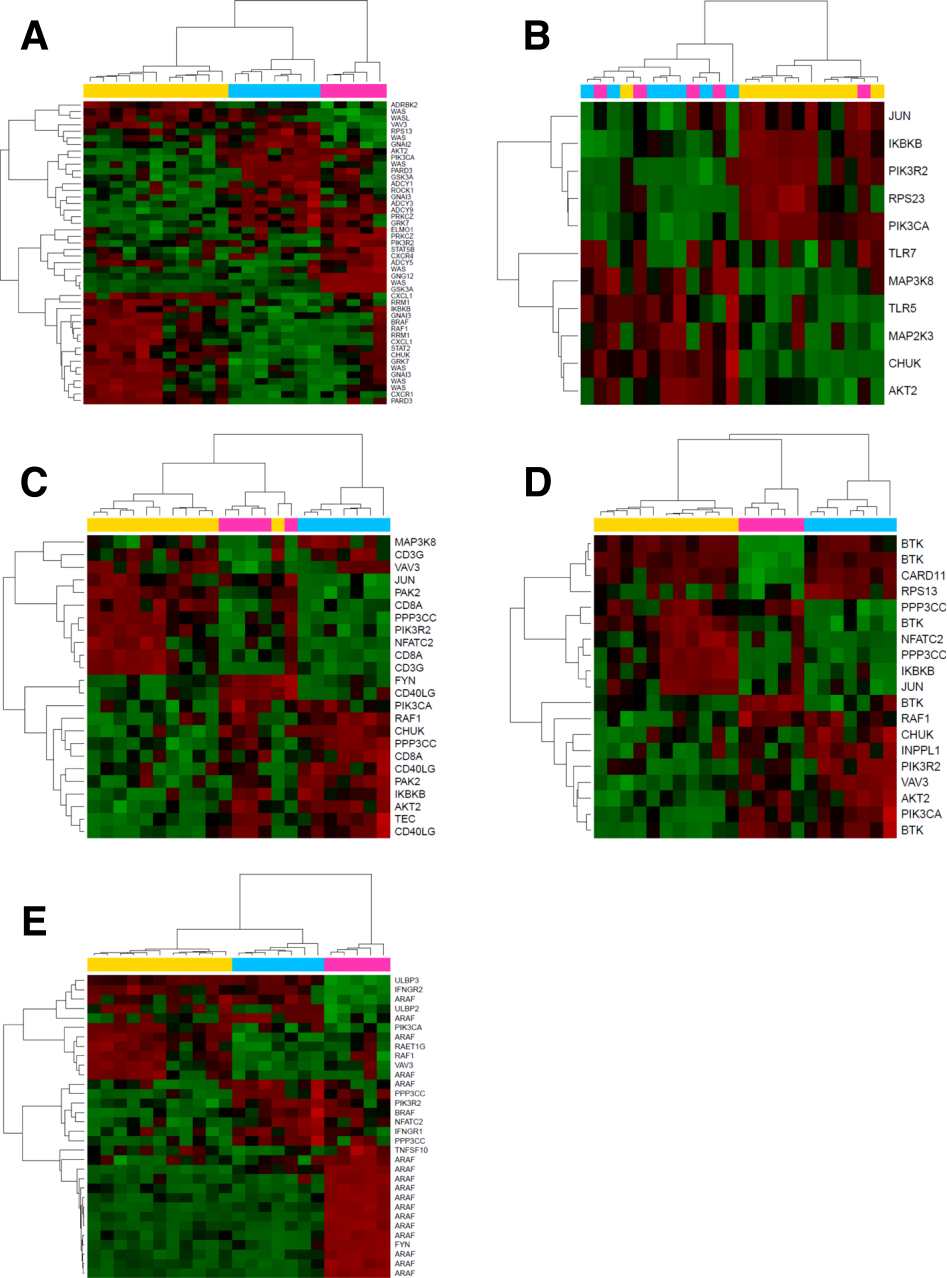
HNC survivors display differential methylation in inflammatory pathways. DMPs were examined in the (**a**) chemokine signaling pathway, (**b**) toll-like receptor (TLR) signaling pathway, (**c**) T-cell receptor signaling pathway, (**d**) B-cell receptor signaling pathway, and E) natural killer cell mediated cytotoxicity pathway. Each row represents one DMP and is labeled with the associated gene name. All reported DMPs have FDR *p*-value >0.05

Inflammation impacts long-term outcomes in cancer survivors, so we focused on immune-related epigenetic changes. We first examined methylation in KEGG pathways involved in inflammatory signaling and immune cell-specific processes: chemokine signaling pathway (hsa04062), toll-like receptor (TLR) signaling pathway (hsa04620), T-cell receptor signaling pathway (hsa04660), B-cell receptor signaling pathway (hsa04662), and natural killer cell-mediated cytotoxicity (hsa04650). We considered gene-associated CpGs that were significantly differentially methylated in at least one GroupWise comparison (FDR adjusted *p* value <0.05). In the chemokine signaling pathway, the three methylation groups displayed distinct patterns, but the yellow and blue groups were more similar to each other than to the pink group (Fig. 2a). In the TLR signaling pathway, two patterns emerged (Fig. 2b). The yellow group was very distinct from the other two groups and notably showed hypomethylation of *TLR5*, component of inhibitor of NFKB kinase complex (*CHUK*), and two mitogen-activated protein kinases (*MAP3K8* and *MAP2K3*). Additionally, Jun proto-oncogene (*JUN*) and inhibitor of NFKB kinase subunit beta (*IKBKB*) were hypermethylated in the yellow group.

Cell type-specific pathways were also investigated. The buffy coat contains a mixture of leukocytes, so differential methylation could provide clues as to the cellular composition and inflammatory state of the HNC survivors. In the T-cell receptor signaling pathway, the three methylation groups had different methylation patterns, but the blue group was more distinct than the other two groups (Fig. 2c). Interestingly, hypermethylation in the blue group occurred at two CpGs associated with CD40 ligand (*CD40LG*) one site associated with Tec protein tyrosine kinase (*TEC*). Nuclear factor of activated T-cells 2 (*NFATC2*) and *CD8A* were hypomethylated in the blue group. The three methylation groups displayed very unique methylation patterns in the B-cell signaling pathway (Fig. 2d). Finally, methylation of genes involved in natural killer cell-mediated cytotoxicity was differentially methylated between the three groups, but the blue and yellow groups were more similar to each other than to the pink group (Fig. 2e). Several CpGs were associated with A-Raf proto-oncogene (*ARAF*) and were hypermethylated in the pink group.

**Fig. 3:**
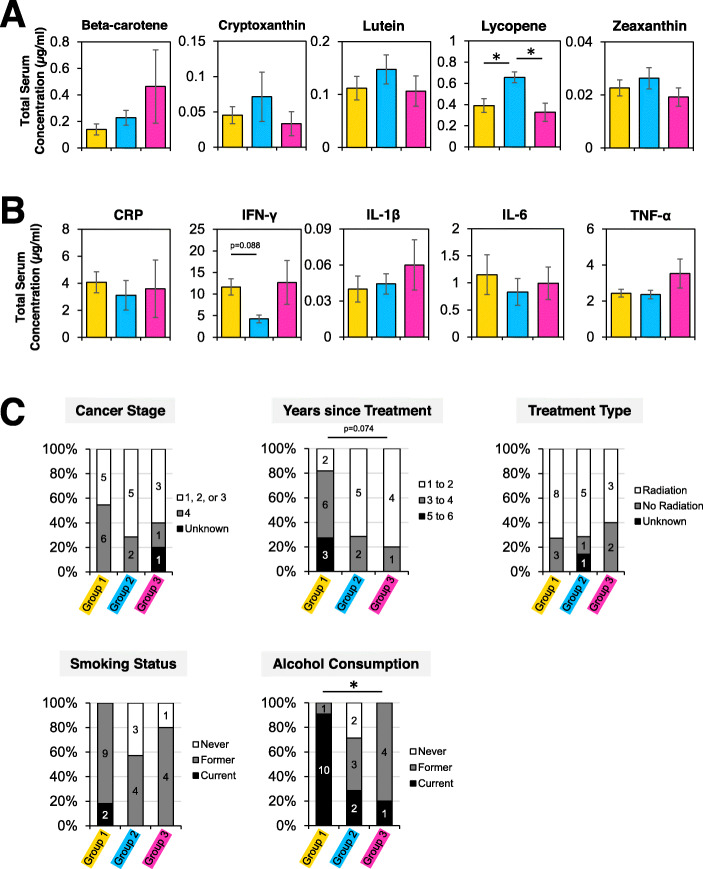
Methylation patterns are reflective of total serum lycopene and alcohol consumption. **a** Circulating carotenoids and **b** cytokines were measured in the three differentially methylated groups. Data are presented as mean ± SEM. *Denotes *p* value <0.05 using posthoc Tukey test. **c** Group differences in lifestyle parameters were assessed using chi-square tests. Y-axes represent the percentage of participants within each category, with the actual number of participants noted on the graph. *Denotes *p* value <0.05 using chi-square test

We then sought to uncover how clinical and lifestyle mediators of inflammation differed between methylation groups. Serum cytokine and carotenoid levels were compared between the three methylation patterns. Two distinct phenotypes emerged based on serum lycopene levels. The blue group had higher lycopene levels than the yellow and pink groups (Fig. 3a). Cytokine levels were similar between all three methylation groups (Fig. 3b). There was a trend for lower IFN-γ levels in the blue versus yellow group (*p* = 0.088).

Next, we compared clinical and lifestyle features between the three methylation groups (Fig. 3c). There was no significant difference in cancer stage, years since treatment, treatment type, or smoking status among the three groups using chi-square tests. However, alcohol consumption varied between groups. The yellow group consisted predominantly of current alcohol drinkers, while the blue and pink groups had more individuals who had never consumed alcohol or had formerly consumed alcohol. Additionally, there was a trend for group differences in years since cancer treatment (*p* = 0.074). The yellow group consisted of individuals who had undergone cancer treatment three or more years prior to participation in the study, whereas the blue and pink groups consisted of individuals who had undergone treatment more recently.

### The blue group of HNC survivors is characterized by differential methylation of genes involved with metabolic, transcriptional, and translational regulation

**Fig. 4:**
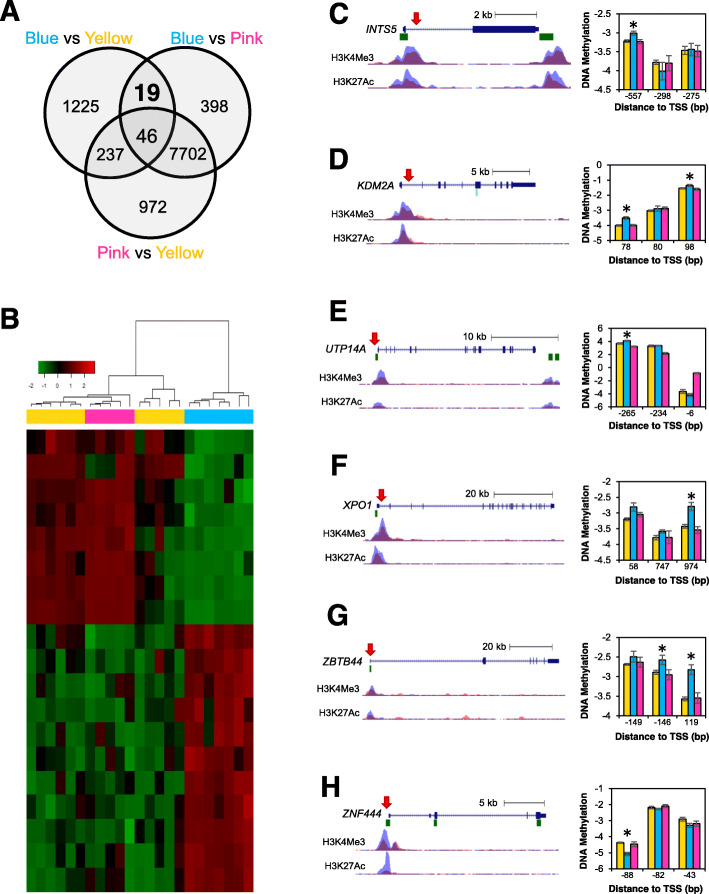
The blue methylation group is characterized by differential methylation of genes involved in transcriptional and translational regulation. **a** The Venn diagram shows the distribution of DMPs between the three differentially methylated groups. The top left circle represents DMPs between the blue and yellow groups. The top right circle represents DMPs between blue and pink groups. The bottom circle represents DMPs between the pink and yellow groups. **b** The heatmap shows the hierarchical clustering of participants using the 19 DMPs that were observed between blue vs yellow and blue vs pink, but not pink vs yellow. The promoters of (**c**) *INTS5*, **d**) *KDM2A*, (**e**) *UTP14A*, (**f**) *XPO1*, (**g**) *ZBTB44*, and (**h**) *ZNF444* were examined for differential methylation. DNA methylation is reported using M-values as mean ± SEM. All reported DMPs have FDR *p* value >0.01

Given that the blue group showed altered methylation in the T-cell receptor signaling pathway as well as high lycopene levels, we then sought to examine highly significant genes that were differentially methylated in the blue group. We selected the unique subset of DMPs that were differentially methylated in the blue vs yellow groups as well as in the blue vs pink groups, but not differentially methylated in the yellow vs pink groups (Fig. 4a). In order to focus on highly robust loci, the FDR adjusted *p* value threshold was set at 0.01. This yielded a small but highly significant set of 19 DMPs that were similarly methylated in the yellow and pink groups, and differentially methylated in the blue group (Fig. 4a, b).

First, we looked at the position of the DMPs relative CpG islands. Islands were defined as regions with a high density of CpGs (observed-to-expected CpG ratio ≥60%). Shores refer to regions within 2 kb upstream or downstream of an island. The open sea is located beyond the 4 kb region flanking CpG islands [35]. In our experiment, 10 of the 19 DMPs were located in CpG islands, five were in shores, and four were in the open sea (Table 1). Five were located in the gene body, four were within the first exon, three were in the 5’ UTR, eight were within 1500 bp of the transcription start site (TSS) of a gene, and two were in an intergenic region. Furthermore, the DMPs were associated with regulatory features, including nine that were in a promoter region and two that were in an enhancer. Of the 19 DMPs, 11 were hypermethylated in the blue group.

**Table 1:** Annotation of differentially methylated probes in the blue group

Probe ID	Gene	Name	Primary function	Relation to island	CpG location	Regulatory feature	Methylation
cg02010442^a^	ATG4A	Autophagy related 4A cysteine peptidase	Autophagy	Island	1^st^ Exon, 5’UTR	Promoter	Hypomethylated
cg02010442^a^	PSMD10	Proteasome 26S Subunit, Non-ATPase 10	Protein degradation	Island	TSS200	Promoter	Hypomethylated
cg03219922	N/A	N/A	N/A	Shore	N/A	N/A	Hypermethylated
cg04621904	FAM47C	Family with sequence similarity 47 member C	Unknown	Shore	1^st^ Exon	None	Hypermethylated
cg06081716	SENP7	SUMO/Sentrin specific peptidase 7	SUMOylation	Shore	TSS1500	Promoter	Hypermethylated
cg07917502^b^	PREPL	Prolyl endopeptidase like	Proteolysis	Island	TSS200	Promoter	Hypermethylated
cg07917502^b^	CAMKMT	Calmodulin-lysine N-methyltransferase	Calcium signaling	Island	TSS200	Promoter	Hypermethylated
cg09130556	CYP1B1	Cytochrome P450 family 1 subfamily B member 1	Cytochrome p450	Island	TSS1500	Enhancer	Hypermethylated
cg11585357	N/A	N/A	N/A	Sea	N/A	Enhancer	Hypomethylated
cg11754259	ZBTB44	Zinc finger and BTB domain containing 44	Transcription	Island	1^st^ Exon, 5’UTR	None	Hypermethylated
cg11873147	C1QL2	Complement C1q like 2	Complement protein	Island	Gene Body	None	Hypermethylated
cg14137558	UTP14A	UTP14A, small subunit processome component	Ribosomal protein	Island	TSS200	Promoter	Hypomethylated
cg15682807	ITM2A	Integral membrane protein 2A	Membrane Protein	Sea	1^st^ Exon	None	Hypomethylated
cg15842967	KDM2A	Lysine demethylase 2A	Transcription, histone modification	Sea	Gene Body	Promoter	Hypermethylated
cg17145281	INTS5	Integrator Complex Subunit 5	Integrator complex with RNA Pol II	Shore	Gene Body	Promoter	Hypermethylated
cg17716100	FOXO4	Forkhead box O4	Transcription factor, lipid metabolism	Island	TSS1500	Promoter	Hypomethylated
cg21888438	MID1IP1	MID1 interacting protein 1	Lipid metabolism	Island	Gene Body	None	Hypomethylated
cg24890023	XPO1	Exportin 1	Nuclear export	Shore	5’UTR	None	Hypermethylated
cg26708638	ZNF444	Zinc finger protein 444	Transcription	Island	TSS200	Promoter	Hypomethylated
cg27360006	ZNF41	Zinc finger protein 41	Transcription	Sea	Gene Body	None	Hypermethylated
cg27609596	PTCHD1	Patched domain containing 1	Membrane protein, autism	Island	TSS1500	None	Hypomethylated

5’UTR: 5’ untranslated region, TSS200: within 200 bp of the transcription start site, TSS1500: within 1500 bp of the transcription start site. ^a^cg02010442 and ^b^cg07917502 were each associated with two different genes.

Additionally, 15 DMPs were associated with exactly one gene, two DMPs were associated with two genes each, and two DMPs were not associated with any gene (Table 1). Three genes were involved in metabolic processes, including autophagy-related 4A cysteine peptidase (*ATG4A*), Forkhead box O4 (*FOXO4*), and MID1 interacting protein 1 (*MID1IP1*). Eight genes were involved in regulation of transcription or translation: *FOXO4*, integrator complex subunit 5 (*INTS5*), lysine demethylase 2A (*KDM2A*), UTP14A small subunit processome component (*UTP14A*), exportin 1 (*XPO1*), zinc finger and BTB domain containing 44 (*ZBTB44*), zinc finger protein 41 (*ZNF41*), and zinc finger protein 444 (*ZNF444*). The remaining genes had either unknown or miscellaneous functions, such as proteolysis (*PREPL*), cytochrome p450 activity (*CYP1B1*), and calcium signaling (*CAMKMT*).

We then examined methylation around transcriptional and translational regulation genes. Six genes contained DMPs within 1 kb of the TSS, including *INTS5*, *KDM2A*, *UTP14A*, *XPO1*, *ZBTB44*, and *ZNF44* (Fig. 4c–h). Only the DMP within *ZNF41* was located downstream in the second intron. We examined the promoter region of the genes to identify any other DMPs within close proximity. Specifically, we examined the region within 1 kb of the TSS. To ensure that the region was indeed a promoter, we used data from ENCODE to identify enrichment of histone 3 lysine 4 trimethylation (H3K4Me3) and histone 3 lysine 27 acetylation (H3K27Ac), which are indicative of active promoters [36]. *KDM2A* and *ZBTB44* each contained two DMPs within the promoter while all others contained one DMP (Fig. 4c–h).

### The yellow group of HNC survivors is characterized by differential methylation of genes encoding histone and mitochondrial ribosomal proteins

**Fig. 5:**
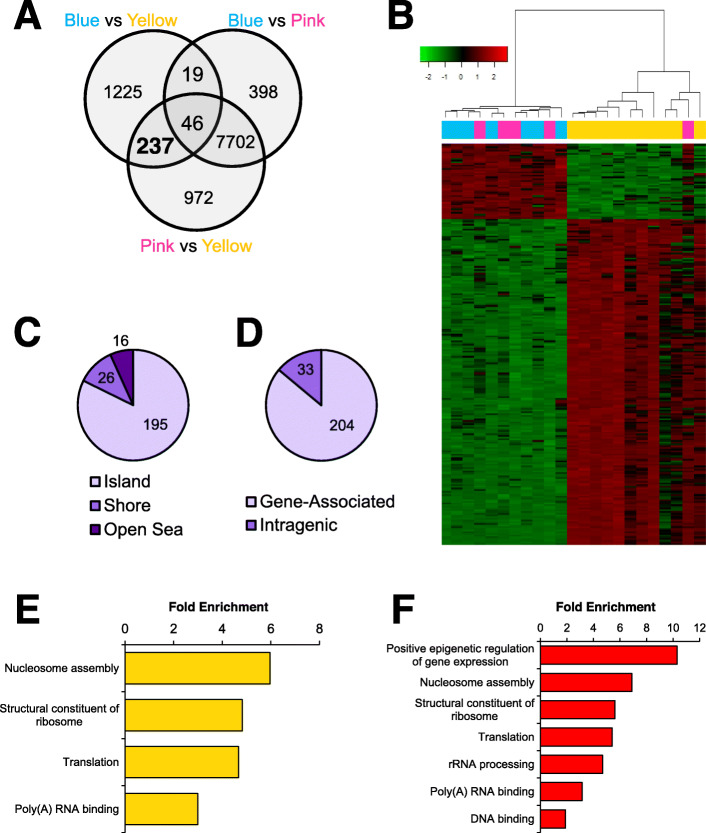
The yellow methylation group is characterized by differential methylation of genes involved in genomic structure and translation. **a** The Venn diagram shows the distribution of DMPs between the three differentially methylated groups. The top left circle represents DMPs between the blue and yellow groups. The top right circle represents DMPs between blue and pink groups. The bottom circle represents DMPs between the pink and yellow groups. **b** The heatmap shows the hierarchical clustering of participants using the 237 DMPs that were observed between yellow vs blue and yellow vs pink, but not blue vs pink. DMPs were associated with genomic features including (**c**) CpG islands and (**d**) coding genes. Gene ontology enrichment analysis was performed on E) all DMPs and F) only hypermethylated DMPs. All reported DMPs have FDR *p*-value >0.01

The yellow methylation group had distinct DNA methylation in the TLR signaling pathway as well as higher alcohol consumption than the other two groups, so we further investigated differential methylation of specific genes in the yellow group. We selected the unique subset of DMPs with an FDR *p* value <0.01 that were differentially methylated in the blue vs yellow groups as well as in the pink vs yellow groups, but not differentially methylated in the blue vs pink groups (Fig. 5a). Overall, 237 DMPs that were similarly methylated in the blue and pink groups, and differentially methylated in the yellow group (Fig. 5a, b). We found that a majority of DMPs were hypermethylated in the yellow group (*n* = 193). All DMPs are detailed in Supplementary file 10.

We then looked at the position of DMPs relative to CpG islands and genes. In our experiment, 195 of the DMPs were located in CpG islands, 26 were in shores, and 16 were in the open sea (Fig. 5c). There were 204 DMPs that were associated with at least one gene and 33 DMPs that were located in an intergenic region (Fig. 5d). Enrichment analysis of all DMPs revealed enrichment of four functions and processes including nucleosome assembly, structural constituent of ribosome, translation, and poly(A) RNA binding (Fig. 5e). When only hypermethylated DMPs were considered, the same four categories were enriched, but additionally, positive epigenetic regulation of gene expression, rRNA processing, and DNA binding were also enriched (Fig. 5f).

**Fig. 6:**
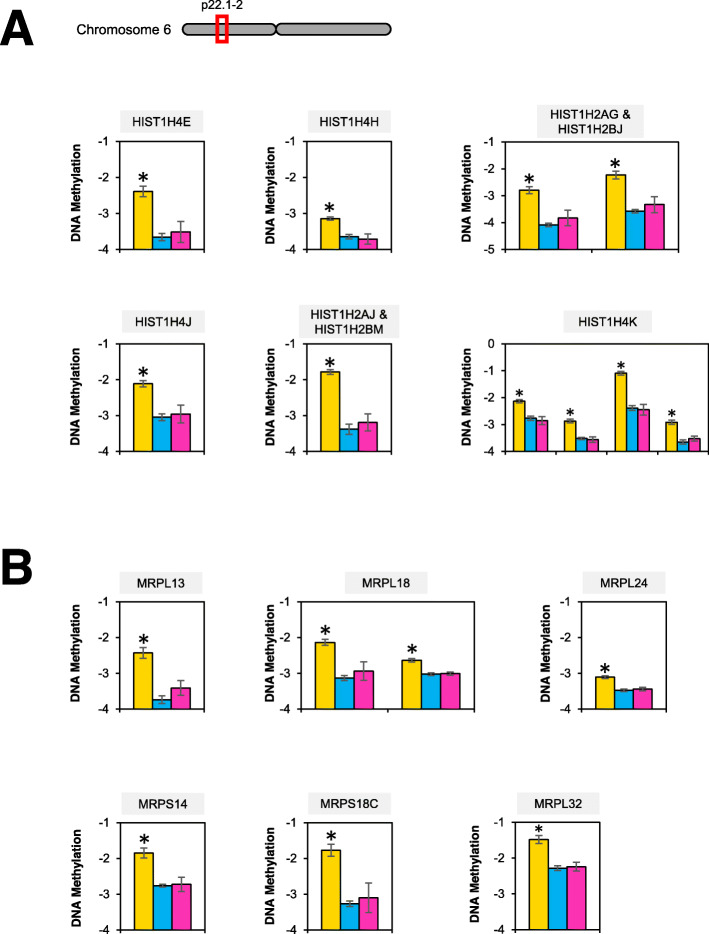
Hypermethylation of genes encoding histone and mitochondrial ribosomal proteins is characteristic of the yellow methylation group. **a** One locus on chromosome 6 contained ten DMPs that were associated with eight distinct genes encoding histone subunits. (**b**) Six mitochondrial ribosomal proteins were hypermethylated in the yellow group. DNA methylation is reported using M-values as mean ± SEM. *Denotes FDR *p*-value <0.01

Finally, we investigated the specific genes within the enriched biological processes. Interestingly, several genes encoding histone proteins were hypermethylated. Several loci were located on the short arm of chromosome 6. There was one hypermethylated DMP in the promoters of *HIST1H4E*, *HIST1H4H*, *HIST1H4J*, *HIST1H2AJ*, and *HIST1H2BM* (Fig. 6a). There were two hypermethylated DMPs in the promoters of *HIST1H2AG* and *HIST1H2BJ*. There were four hypermethylated DMPs in the promoter of *HIST1H4K*. Genes encoding mitochondrial ribosomal proteins (MRPs) were also hypermethylated. There was one hypermethylated DMP in the promoters of *MRPL13*, *MRPL24*, *MRPS14*, *MRPS18C*, and *MRPL32* (Fig. 6b). There were two hypermethylated DMPs in the promoter of *MRPL18*.

## Discussion

In this study, DNA methylation profiles in leukocytes were used to stratify HNC survivors into three distinct groups. Each group displayed a unique methylation signature in inflammatory pathways. One group of survivors was defined by high lycopene levels, distinct methylation in the T-cell receptor signaling pathway, and hypermethylation of transcriptional and translational regulators. Another group exhibited distinctive methylation in the toll-like receptor (TLR) signaling pathway, hypermethylation of mitochondrial ribosomal genes, and higher rates of alcohol consumption.

In the present study, we found one group of HNC survivors that was characterized by high lycopene levels and hypermethylation of transcriptional and translational mediators as well as T-cell signaling genes. These findings might point to lower levels of oxidative damage and reduced T-cell activation. T-cell activation occurs when an antigen and costimulatory factor are recognized, inducing cytoskeleton reorganization, cytokine release, and proliferation. Activated T-cells can then present an antigen and a costimulator to B-cells to induce class switching. We found hypermethylation of key T-cell signaling proteins, such as *TEC*, *IKBKB*, and the costimulatory factor, *CD40LG*, suggesting lower T-cell activation. We also identified the hypermethylation of transcription factors, which could alter the processes needed for cellular proliferation. Additionally, two hypomethylated DMPs were associated with *CD8A*, which could point to altered levels of CD8+ T-cells. Not only are low levels of circulating CD8+ T-cells characteristic of HNC patients, but low levels of CD8+ T-cells have also been correlated with poor survival outcomes [37, 38]. Because we saw hypomethylation of *CD8A*, we might expect a higher expression of CD8 and more CD8+ T-cells. Future experimentation should evaluate the relationship between DNA methylation and white blood cell composition to assess any differences in long-term outcomes.

The relationship between lycopene and DNA methylation has not been thoroughly described. One study in obese adults found an inverse correlation between lycopene intake and methylation of a CpG in the paraoxonase 1 (*PON1*) promoter [39]. An in vitro study observed demethylation of the glutathione S-transferase pi 1 (*GSTP1*) promoter in lycopene-treated MDA-MB-468 breast cancer cells [4]. The same study reported demethylation of retinoic acid receptor β (*RARB2*) and secretoglobin family 3A member 1 (*SCGB3A1*) in noncancerous breast cancer cells following lycopene treatment. These results were not reproducible in prostate cancer cells [40, 41]. In addition to direct lycopene-induced DNA methylation, lycopene may exert systemic antioxidant effects that alter DNA methylation. Oxidative markers were not measured in this study; however, previous experimentation has shown that serum lycopene levels are negatively correlated with oxidative stress [42].

The impact of oxidative stress and DNA methylation is an active area of research. In a cohort of 966 elderly men and women, microarray analysis of blood samples revealed 66 CpGs that were associated with at least one marker of oxidative stress [43]. Similarly, in biliary atresia patients, LINE-1 methylation was inversely correlated with plasma 8-hydroxy-2′-deoxyguanosine (8-OHdG), a marker of oxidative DNA damage. Additionally, oxidative stress was found to be directly correlated with malignancy grade and inversely correlated to global DNA methylation in glioma tumor samples [44]. In a cell model, exposure to the reactive oxygen species hydrogen peroxide (H_2_O_2_) upregulated DNA demethylating enzyme MBD4 and promoted cell survival [45]. The mechanism mediating oxidative stress-induced DNA methylation has been hypothesized to involve cofactors for DNA methyltransferases and demethylases. Niu et al. explored this hypothesis by showing that cells exposed to H_2_O_2_ had a reduced GSH/GSSG ratio, higher DNA methylation levels, and lower TET activity. Because the TET protein uses Fe^++^ as a cofactor for the demethylation reaction, the authors concluded that reactive oxygen species oxidize Fe^++^ to Fe^+++^ to limit cofactor availability and prevent demethylation [46].

We also identified a group of HNC survivors that was characterized by high rates of alcohol consumption, hypermethylation of histone and mitochondrial ribosomal genes, and distinct methylation in the TLR signaling pathway. These findings point to elevated inflammation and greater potential for NFKB-mediated cytokine effects in this subset of survivors. Previous reports have found an association between alcohol intake, systemic inflammation, and risk of HNC. Alcohol consumption is correlated with higher HNC incidence and poor survival [47–50]. Additionally, alcohol has been shown to increase gut permeability [51], elevate circulating lipopolysaccharide (LPS) levels [52], and alter white blood cell composition [53]. Our findings suggest that high alcohol intake is also associated with changes in DNA methylation. Alcohol is thought to alter folate absorption and impair one-carbon metabolism and has been shown to induce global tumor hypomethylation in HNC [54]. Alcohol consumption has also been associated with altered DNA methylation in leukocytes [55, 56]. We showed hypermethylation of leukocyte mitochondrial ribosomal proteins in a group of HNC survivors with high rates of alcohol intake. Mitochondrial ribosomes translate proteins involved in oxidative phosphorylation; however, mitochondrial ribosomal proteins also participate in apoptosis [57]. For instance, MRPS29 and MRPL41 have been shown to activate caspase-mediated apoptosis and inhibit tumor growth [58, 59]. Given the assumption that promoter methylation suppresses gene expression, we hypothesize that the methylation changes observed in our study may indicate lower levels of oxidative phosphorylation and apoptosis in leukocytes. We also found specific methylation changes in TLR signaling genes. Specifically, we found hypomethylation of *TLR5* and *CHUK*, suggesting higher expression and greater potential for activation of NFKB and proinflammatory effects. In this study, we only show an association between serum alcohol and DNA methylation and make interpretations about their impact on inflammatory processes, but future work should test these relationships for causality and functional outcomes.

Although our study is limited by the small sample size, HNC is a rare and understudied disease. This study contributes to the limited literature on HNC by identifying novel epigenetic associations and provides several avenues for further exploration. The analysis is somewhat limited by the 450 k array because while it covers functionally relevant CpGs in promoters, enhancers, and regions with high CpG density, it excludes many isolated CpGs. Nonetheless, the 450 k array has been used repeatedly for DNA methylation analysis and is able to extensively cover CpGs with probable regulatory functions. Future experimentation may examine how DNA methylation regulates the expression of the identified genes and whether changes in the epigenome impact quantity and quality of blood cell counts. Furthermore, understanding the functional roles of these genes in immune cells will elucidate the influence they may bear in tumor recurrence and patient survival.

In the present study, HNC survivors were stratified based on DNA methylation profiles in leukocytes. Three distinct groups were defined by unique methylation signatures in inflammatory pathways. The groups also differed in their clinical and lifestyle characteristics. One group of survivors had high lycopene levels, distinct methylation in the T-cell receptor signaling pathway, and hypermethylation of transcriptional and translational regulators. Another group had distinctive methylation in the toll-like receptor (TLR) signaling pathway, hypermethylation of mitochondrial ribosomal genes, and higher rates of alcohol consumption. Our study provides insight into the variable inflammatory and epigenetic landscapes in HNC survivors. Future investigation of DNA methylation, inflammation, and long-term outcomes in HNC survivors will elucidate potential strategies for maximizing health and quality of life in HNC survivors.

## Supplementary information

**Additional file 1.** DMPs between blue and pink groups.

**Additional file 2.** DMRs between blue and pink groups.

**Additional file 3.** DMPs between blue and yellow groups.

**Additional file 4.** DMRs between blue and yellow groups.

**Additional file 5.** DMPs between pink and yellow groups.

**Additional file 6.** DMPs between pink and yellow groups.

**Additional file 7.** Gene ontology enrichment analysis in the blue group.

**Additional file 8.** Gene ontology enrichment analysis in the pink group.

**Additional file 9.** Gene ontology enrichment analysis in the yellow group.

**Additional file 10.** DMPs in the yellow group.

## Data Availability

The datasets used and/or analyzed during the current study are available from the corresponding author on reasonable request.

## Abbreviations

HNC: Head and neck cancer
CD40LG: CD40 ligand
TEC: Tec protein tyrosine kinase;
TLR: Toll-like receptor
IL-6: Interleukin 6
CRP: C-reactive protein
HPV: Human papillomavirus
UAB: University of Alabama-Birmingham
UIUC: University of Illinois Urbana-Champaign
IFN- γ: Interferon-gamma
IL-1β: Interleukin-1 beta
TNF-α: Tumor necrosis factor alpha
FDR: False discovery rate
DMP: Differentially methylated probes
DMR: Differentially methylated regions
CENP-A: Centromere protein A;
SRP: Signal recognition particle
hsa04062: chemokine signaling pathway
hsa04620: toll-like receptor signaling pathway
hsa04660: T-cell receptor signaling pathway
hsa04662: B-cell receptor signaling pathway
hsa04650: natural killer cell mediated cytotoxicity
JUN: Jun proto-oncogene
NFATC2: Nuclear factor of activated T-cells 2
ARAF: A-Raf proto-oncogene
ATG4A: Autophagy-related 4A cysteine peptidase
FOXO4: Forkhead box 04
MID1IP1: MID1 interacting protein
INTS5: Integrator complex subunit 5
KDM2A: Lysine demethylase
UTP14A: UTP14A small subunit processome component
XPO1: Exportin 1
ZBTB44: zinc finger and BTB domain containing 44
ZNF41: zinc finger protein 41
ZNF444: zinc finger protein 444
PREPL: proteolysis
CYP1B1: Cytochrome p450 activity
CAMKMT: Calcium signaling
H3K4Me3: Histone 3 lysine 4 trimethylation
H3K27Ac: Histone 3 lysine 27 acetylation
PON1: Paraoxonase 1
GSTP1: Glutathione S-transferase pi 1
RARB2: Retinoic acid receptor β
SCGB3A1: Secretoglobin family 3A member
8-OHdG: Plasma 8-hydroxy-2′-deoxyguanosine
H_2_O_2_: hydrogen peroxide
